# Network analysis of gene essentiality in functional genomics experiments

**DOI:** 10.1186/s13059-015-0808-9

**Published:** 2015-10-30

**Authors:** Peng Jiang, Hongfang Wang, Wei Li, Chongzhi Zang, Bo Li, Yinling J. Wong, Cliff Meyer, Jun S. Liu, Jon C. Aster, X. Shirley Liu

**Affiliations:** Department of Biostatistics and Computational Biology, Dana-Farber Cancer Institute, Harvard T.H. Chan School of Public Health, Boston, MA 02215 USA; Department of Pathology, Brigham and Women’s Hospital, Boston, MA 02115 USA; Department of Statistics, Harvard University, Cambridge, 200092 China; School of Life Science and Technology, Tongji University, Shanghai, MA 02138 USA

**Keywords:** CRISPR screen, Network analysis, Gene essentiality

## Abstract

Many genomic techniques have been developed to study gene essentiality genome-wide, such as CRISPR and shRNA screens. Our analyses of public CRISPR screens suggest protein interaction networks, when integrated with gene expression or histone marks, are highly predictive of gene essentiality. Meanwhile, the quality of CRISPR and shRNA screen results can be significantly enhanced through network neighbor information. We also found network neighbor information to be very informative on prioritizing ChIP-seq target genes and survival indicator genes from tumor profiling. Thus, our study provides a general method for gene essentiality analysis in functional genomic experiments (http://nest.dfci.harvard.edu).

**Electronic supplementary material:**

The online version of this article (doi:10.1186/s13059-015-0808-9) contains supplementary material, which is available to authorized users.

## Background

Essential genes are those genes critical for cell viability under certain contexts. Recent years have seen the rapid development of functional genomics techniques for studying gene essentiality genome-wide. For example, large-scale shRNA screens have been used to search for essential genes in diverse cell lines [1]. If a specific transcription factor drives the cell viability under certain condition, ChIP-seq technique can be used to profile the regulatory targets to further find essential genes [2]. Many computational methods have also been developed to predict context specific gene essentiality through integration of gene expression, molecular alterations, and biological pathways [3].

Recently, the CRISPR (clustered regularly interspaced short palindromic repeats) screen emerged as an exciting new approach to profile gene essentiality at genome scale [4–11]. In the CRISPR system, single-guide RNAs (sgRNA) direct Cas9 nucleases to induce double-strand breaks (DSB) at targeted genomic regions [12, 13]. When the error-prone non-homologous end-joining mechanism repairs the DSBs, insertions and deletions occur with high frequency, which produce a non-functional protein. Catalytically inactive Cas9 fused with a transcriptional activator or repressor has also been used to modulate gene expression at targeted loci [8, 9, 14–17]. Combined with lentiviral delivery method, CRISPR systems enable genome-scale functional screening in a cost-effective manner [4–11]. In CRISPR screens, sgRNAs targeting candidate genes are synthesized, and viral integration enables readout through next-generation sequencing [18]. The relative abundances of each integrated sgRNA between different conditions are compared and the importance of sgRNA target gene is inferred according to its sgRNAs’ effect on cell growth.

The progress of CRISPR screen technology enabled systematic and reliable determination of gene essentiality under diverse conditions. The high quality gene essentiality profiles from CRISPR could enable a better comparison among essentiality prediction methods and better identification of distinct features of the essential genes. Such features not only facilitate a better understanding of the CRISPR screen data, but also can help prioritize the leads from CRISPR screens. From the analysis of yeast protein interactions, it is well known that highly connected proteins in a network (degree hubs) are more likely to be essential for viability [19–21]. Thus, we hypothesize that the gene essentiality outcome in CRISPR screens might depend on the gene connectivity in biological networks. Protein interaction networks have been integrated to improve the quality of RNAi screen results, which are very noisy due to off-target effect and low knockdown efficiency [22–25]. These previous works on RNAi screen indicate that the CRISPR screen result quality may also be improved by integration with protein interaction networks.

In this study, we took a network perspective and developed a method called NEST (Network Essentiality Scoring Tool) to systematically analyze the recent genome wide CRISPR screen data. We found that gene essentiality determined by CRISPR screen largely depends on the expression level of interacting genes in the biological network. Moreover, the quality of CRISPR and shRNA screen data can be further improved by NEST after considering the gene neighborhood screen outcome. Besides applications on CRISPR and shRNA screens, NEST is also generally applicable on many other types of genomics data analysis, such as ChIP-seq target gene prioritization and survival gene identification from tumor profiling data.

## Results and discussion

### NEST predicts gene essentiality in CRISPR screen

We first collected recently published CRISPR loss-of-function screen data [4, 5, 8], and selected three cell lines (K562, HL60, and A375) with publicly available gene expression data [26–28]. The significant CRISPR screen gene hits are called with software MAGeCK [29]. In CRISPR screens for growth phenotype, most significant genes are negatively selected, which means these genes are essential in the corresponding experimental condition (Additional file 1: Figure S1). To identify distinct features of gene essentiality in CRISPR screens, we developed a network-based method called NEST (Network Essentiality Scoring Tool), and found the following metric to give reliable performance.

**Fig. 1:**
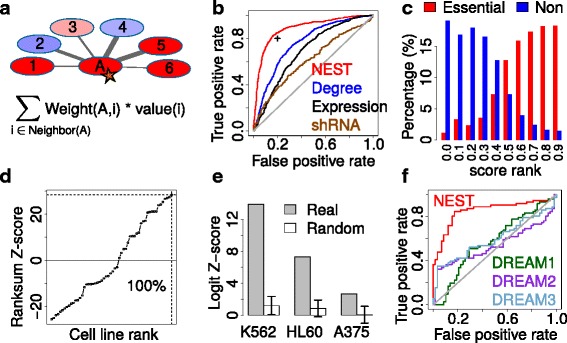
Prediction of CRISPR screen outcome. **a** NEST calculates the neighbor expression of a gene as the sum of expression values of its neighbor genes connected in the network, weighted by the interaction weight. **b** Receiver operating characteristic (ROC) curve is used to test the performance of predicting gene essentiality determined by K562 CRISPRi screen. The performance of NEST score, network degree, gene expression, and shRNA screen are shown. The black point represents false positive rate 0.2 and true positive rate 0.8. **c** The NEST scores are converted to rank percentiles from 0 to 1, and shown for essential genes and non-essential genes determined in K562 CRISPRi screen. **d** For each Roadmap expression profile, we calculated the prediction power of NEST score on gene essentiality in K562 screen by Wilcoxon rank-sum test. The rank-sum Z-scores for all cell lines are ranked and the K562 profile has the largest value. **e** The STRING network was randomized 1,000 times, and the NEST scores were calculated for random networks. We used multivariate logistic regression to test the association of NEST score with gene essentiality after controlling the effects of network degree and gene expression (Table 1). The Logit Z-scores are shown for real and random networks. **f** In DREAM gene essentiality prediction challenge, A375 cell line also has CRISPR screen data available. Using essential genes selected in CRISPR screen as gold standard, the prediction performance is compared between NEST (red) and the top three winners in DREAM

For each gene, NEST calculates neighbor expression measure as the sum of normalized expression of its neighbor genes connected in the protein interaction network, weighted by the interaction confidence (Fig. 1a). The calculation of NEST score can also be formulated as product between connectivity matrix, which is composed of interaction weights between protein pairs, and gene expression vector. Each gene’s relative expression in one cell is normalized against its average expression across all cell lines, and the protein interaction network information is from STRING [30] (Additional file 1: Figure S2). For essential genes selected by CRISPR screen, we defined the gold standard set as the genes hits called by MAGeCK with FDR threshold 0.05 [29]. For each measure, such as NEST score or network degree, all genes were ranked by their values in descending order. Receiver operating characteristic (ROC) curve was used to test the performance of predicting the CRISPR screen gold standard set (Fig. 1b).

For gene essentiality prediction in K562 CRISPRi screen, NEST achieved a false positive rate of 0.2 and a true positive rate of 0.8, with an area under the ROC curve (AUC) of 0.89. The AUC of NEST score is consistently better than network degree, gene expression, and shRNA screen data from the Achilles project [1] (Delong *P* value <1e-10 for all comparisons). Similar performance differences were also observed in CRISPR screen in HL60 and A375 (Additional file 1: Figure S3a). To visualize the CRISPR prediction performance in an intuitive way, we plotted the rank percentile of NEST scores for essential genes and non-essential genes in CRISPR screen (Fig. 1c and Additional file 1: Figure S3B). The NEST ranks are significantly higher for essential genes than non-essential genes (Wilcoxon rank-sum *P* value <1e-10 for cell lines). Besides STRING network, we also used other large-scale networks for CRISPR outcome prediction. However, we did not find any performance improvement using either other network or merged network among several data sources (Additional file 1: Figure S4).

The results above suggest that if a gene’s network neighbors are over-expressed in some conditions, the gene itself becomes more essential. We also found that genes with high NEST scores are tightly clustered in protein interaction network. The STRING network genes were grouped into 2,271 dense complexes using SPICi [31]. Gene with high NEST scores tend to stay in fewer number of STRING clusters than clusters with gene names shuffled (Additional file 1: Figure S5). Thus, a high NEST score may indicate the gene to be member of an active protein complex.

To test the prediction specificity, we applied NEST for gene expression profiles of 56 cell lines profiled by Roadmap project [26]. Measured by rank-sum test Z-scores, K562 CRISPRi screen data achieved the highest association with NEST score in K562 cell than all other cell lines (Fig. 1d). Similarly, HL60 and A375 CRISPR screen data also achieved higher associations with NEST scores in the same cell line (Additional file 1: Figure S3C). Housekeeping genes, such as ribosome members are often selected as essential genes in CRISPR screens [5, 8]. Thus, we further tested that the high prediction power of NEST scores was not purely derived from the same set of housekeeping genes. The prediction performance of NEST remains high after removal of housekeeping genes annotated previously [32] (Additional file 1: Figure S6). Notably, the majority of essential genes selected in CRISPR screen do not overlap between K562, HL60, and A375 (Additional file 1: Table S1). Thus, our NEST score is an orthogonal feature of CRISPR selected gene essentiality other than the universal housekeeping genes shared across conditions.

Since gene network degree and gene expression are also predicative of gene essentiality (Fig. 1b), we then tested whether the prediction performance of NEST is simply an additive effect of network degree and gene expression (Table 1). Using the gene essentiality in CRISPR screen as responsive variable, we did a multivariate logistic regression among all three covariates (NEST score, gene network degree, and expression). While all covariates are predictive of gene essentiality jointly, NEST has the largest statistical significance defined as Logit Z-score (Table 1). Moreover, the logistic regression fitted value, combining all covariates together, did not improve the CRISPR prediction performance comparing to NEST score alone (Additional file 1: Figure S7). As a further control, we randomized the STRING network but preserved the network degree for each gene [33]. The Logit Z-scores for the NEST score in random networks are significantly smaller than in real data (Fig. 1e, empirical *P* value <0.001 for K562 and HL60, and *P* value = 0.003 for A375).

**Table 1:** Confounding factors for NEST prediction performance

Covariate	Coefficient	Standard error	*Z-*score	*P* value
NEST	0.02329	0.001748	13.32	1.72e-40
Degree	0.00415	0.000846	4.91	9.33e-07
Expression	0.12937	0.054223	2.39	1.70e-02
A. K562				
NEST	0.03494	0.00505	6.91	4.73e-12
Degree	0.00608	0.00146	4.16	3.13e-05
Expression	0.33873	0.16595	2.04	4.12e-02
B. HL60				
NEST	0.07296	0.02483	2.94	0.00329
Degree	0.00792	0.00357	2.22	0.02647
Expression	1.12266	0.48343	2.32	0.02022
C. A375				

The prediction power of NEST score on gene essentiality decided by CRISPR screen is tested through logistic regression with gene network degree and gene expression as covariates. The Logit Z-score is defined as Coef/Stderr. The *P* value is calculated by Ward test. The result is shown for (A) K562, (B) HL60, and (C) A375

There have been many previous methods developed for gene essentiality prediction. Since CRISPR screen measures the gene essentiality, any previous methods can be predictive for CRISPR outcome. In a recent DREAM challenge, contenders were asked to develop algorithms to predict cell specific gene essentiality [3]. Among cell lines included in the DREAM challenge, A375 has CRISPR screen data available. We compared the CRISPR outcome prediction performance between our method and the top three methods from the DREAM challenge, and found NEST to consistently outperform all DREAM winners (Fig. 1f). Besides the methods in DREAM, we also compared the performance of NEST with other methods using gene expression and network to predict gene essentiality [34, 35]. NEST significantly outperformed all other methods (Additional file 1: Figure S8 and Additional file 1: Methods).

Besides gene expression, we also used H3K27ac histone mark data to compute NEST scores and tested the gene essentiality prediction performance. Previously, we developed a method to calculate the regulatory potential (RP) scores of a histone modification on each gene promoter from the ChIP-seq profile [36, 37]. Based on our previous method, gene level RP scores in K562 cell were computed using the Roadmap H3K27ac ChIP-seq profile [26]. For each gene, NEST computed neighbor H3K27ac score as the sum of H3K27ac RP scores of its neighbor genes connected in the protein interaction network, weighted by the interaction confidence (Fig. 1a). H3K27ac NEST scores could also reliably predict the gene essentiality in K562 CRISPRi screen (Additional file 1: Figure S3), suggesting the applicability of NEST analysis on histone modification data.

### NEST enhances the quality of CRISPR screen results

**Fig. 2:**
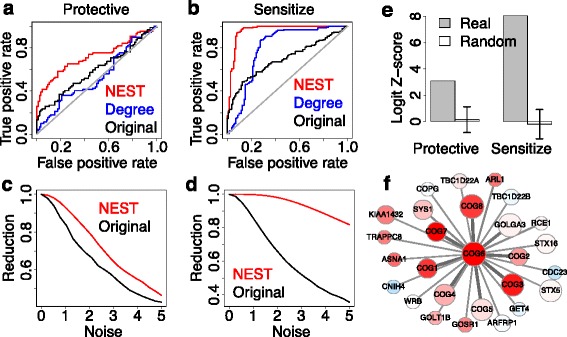
Enhancement of CRISPR screen result. **a** For K562 CRISPRi screen under Cholera/Diptheria toxin selection, the gold standard of toxin protective genes comes from KEGG pathways ‘Vibrio Cholera Infection’ and ‘Glycosphingolipid biosynthesis’. For each gene, NEST computes the neighbor CRISPR score as the sum of CRISPR screen fold change scores of neighbor genes connected in the network. The prediction performance is compared between NEST and original CRISPR scores. **b** The gold standard of toxin sensitizing genes comes from KEGG pathways ‘Ribosome’ and ‘Proteasome’. The prediction performances of NEST and original CRISPR values are compared. **c** The original CRISPR values were randomized by Gaussian white noise. The standard deviation of all original CRISPR values was used as base level. At each noise level relative to the base level, the area under ROC curve (AUC) of prediction is compared with the initial AUC for toxin protective genes in K562 Cholera toxin screen. The reduction ratios were plotted for NEST and original CRISPR scores. **d** The reduction ratios were plotted for toxin sensitizing genes. **e** The STRING network was randomized and the NEST scores were calculated for 1,000 random networks. We used multivariate logistic regression to test the association of NEST scores with gold standards, after controlling the effects from network degree and original CRISPR score. The Logit Z-scores are shown for real and random networks. **f** As an example of high NEST score gene, *COG6* is a component of Golgi complex, and connected with several other components in Golgi complex. The thickness of each edge represents the interaction weight. The color of each gene represents the CRISPR screen fold change score; red color indicates toxin protective and blue color indicates toxin sensitizing

Since early CRISPR screens might have inefficient sgRNA selection and few sgRNA per gene, these screens may not give very strong hits. Encouraged by the prediction performance, we checked whether the network neighbor information could enhance the quality of CRISPR screen results. To measure the quality of a screen data, we need to know the expected outcome. In a K562 CRISPRi screen, the authors performed a genome-scale selection for genes that modulate sensitivity to Cholera/Diphtheria toxin [8]. For genes that work with the toxin, their knock out will protect the cell against the toxin and induce a positive gene selection in screen. For genes that are targeted by toxin, their knock out will sensitize the cell for toxin effect and induce a negative gene selection. The positively selected genes, which played a protective role against toxin, were enriched in KEGG pathways ‘Vibrio Cholerae Infection’ and ‘Glycosphingolipid Biosynthesis’ [8]. The negatively selected genes, which played a sensitizing role for toxin, were enriched in ‘Ribosome’ and ‘Proteasome’ pathways [8]. We used these enriched pathway genes as gold standard and tested how well network interaction could improve the CRISPR screen result (Fig. 2).

For each gene, NEST calculated a neighbor CRISPR score by adding up the CRISPR fold change scores among neighbor genes connected in the STRING network, weighted by the interaction confidence. This NEST score is significantly more predictive on the gold standard outcome than the original CRISPR scores for both protective and sensitizing genes (Fig. 2a, b, Delong test *P* value = 0.010 for protective genes, *P* value = 9.92e-14 for sensitizing genes). Moreover, when we put different levels of Gaussian white noise into CRISPR screen scores, the prediction performance of NEST score diminishes slower than original CRISPR scores (Fig. 2c, d). As a control, if we calculated the NEST scores from randomized network, the prediction power became significantly worse (Fig. 2e, *P* value <0.001 for both protective and sensitizing genes). Thus, through the connectivity of protein interaction network, NEST can enhance the quality of CRISPR screen result.

As an example of gene with high NEST score, *COG6* is a member of Golgi complex and its NEST score is significantly larger than expected (permutation test *P* value <0.001). *COG6* is connected with many other members of Golgi complex (Fig. 2f), and most of them have positive CRISPR screen fold change scores. Since they are connected with each other in network, they mutually boosted each other’s NEST scores. Our result is consistent with the knowledge that cholera toxin needs to enter host cells and travel through the trans-Golgi network to take effect [38].

The above results suggest that if a gene’s network neighbors are under CRISPR screen selection, the gene itself is more likely to be under CRISPR screen selection in the same direction. Besides CRISPR screen, we applied NEST on the Achilles shRNA screen data [1]. NEST can also significantly enhance the quality of shRNA screen result (Additional file 1: Figure S9). Thus, in general, the quality of functional genomic screen result can be improved by considering the gene network neighbor information.

Previously, there were methods developed to improve the quality of RNAi screen results from integration with protein interaction networks [25]. For CRISPR enhancement, we compared our method NEST against NePhe, which was a leading method on RNAi screen network analysis [24]. Using K562 toxin screen as the gold standard, we found that NePhe and NEST show similar performance as measured by ROC curves (Additional file 1: Figure S10AB). However, while NePhe used 14 GB memory and 6.2 h running time, NEST only used 8.3 MB memory and 10.8 s (Additional file 1: Figure S10C). Thus, NEST maintains reliable screen enhancement performance of previous method with much better computational efficiency.

### NEST prioritizes ChIP-seq essential targets

Besides functional genomic CRISPR/shRNA screen, many other genomic experimental techniques can be used to search for essential genes. For example, if a specific transcription factor (TF) drives the cell viability under certain condition, ChIP-seq technique can be used to profile its regulatory targets to further find essential genes [2]. The previous analyses demonstrate that NEST can identify the essential genes in a CRISPR screen. We further explored whether NEST can help prioritize key target genes in a ChIP-seq experiment. ChIP-seq often finds tens of thousands in vivo binding sites for a TF. Since target genes can be regulated by TF binding through long range DNA looping, often thousands of genes near the TF binding sites can be putative targets, and it is hard to prioritize the functional target genes directly from a ChIP-seq experiment. We therefore investigated using network neighbor information to prioritize the functional TF target genes.

**Fig. 3:**
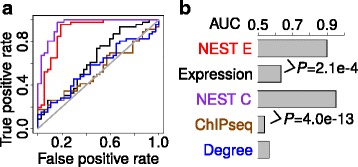
Prediction of NOTCH signaling pathway members. **a** The differential gene expression between *NOTCH* on and off conditions is used to calculate the NEST E score. The NOTCH1 ChIP-seq regulatory potential score for each gene is used to calculate the NEST C score. The KEGG Notch Signaling pathway members are used as gold standard and the prediction performances of all measures are shown by ROC curves. **b** The area under ROC curve (AUC) is shown for each measure. The comparison between AUCs is done by Delong test

Our previous studies of NOTCH1 ChIP-seq and gene expression profiles in the T-lymphoblastic leukemia (TLL) cell line CUTLL1 identified 1,012 differential NOTCH1 binding sites between the *NOTCH* on and off conditions [2]. Based on the ChIP-seq peaks, we calculated a regulatory potential (RP) score for each gene [36, 37, 39], a distance-weighted sum of binding sites measuring the overall regulatory impact of differential NOTCH1 binding on target genes. We set the KEGG NOTCH signaling pathway members as the gold stand, and tested the prediction performance of expression, ChIP-seq RP and NEST scores (Fig. 3). In addition to NEST scores computed from gene expression (NEST E), we also computed NEST scores from ChIP-seq (NEST C), which measures the sum of ChIP-seq RP scores of neighbor genes connected in network. While expression and ChIP-seq measures are barely better than random, NEST scores can predict the annotated KEGG NOTCH signaling pathway members at AUC 0.90 and 0.95 (Fig. 3). It suggests that if a gene’s network neighbors are enriched in the binding target of a TF, the gene itself is more likely to be regulated by the same TF.

### NEST predicts cancer patient survival

**Fig. 4:**
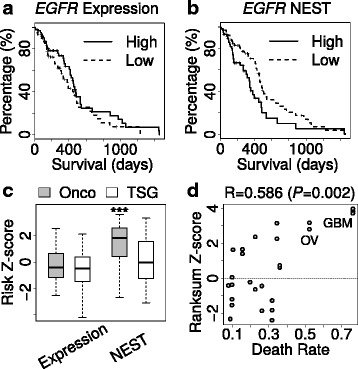
Prediction of patient survival. **a** All TCGA Glioblastoma (GBM) patients are ranked by *EGFR* expression; the top half patients are assigned as high group and the lower half are assigned as low. Kaplan Meier (KM) survival plot is shown for two groups. **b** The survival analysis is done in the same way as A for *EGFR* NEST scores. **c** For each gene, we calculated a death risk Z-score by Cox-PH model from either gene expression or NEST score. We compared the Z-scores for oncogenes (Onco) and tumor suppressor genes (TSG) based on the annotation from Vogelstein et al. The bottom and top of the boxes are the 25th and 75th percentiles (interquartile range). Whiskers on the top and bottom represent the maximum and minimum data points within the range represented by 1.5 times the interquartile range. The distribution of Z-scores is compared by Wilcoxon rank-sum test and three stars represent *P* value <0.001. **d** For each TCGA cohort, the difference of risk Z-scores computed from NEST was tested by Wilcoxon rank-sum test. The rank-sum Z-scores are plotted against the death rate of each cancer type, with Spearman’s rank correlation as title

Encouraged by the above analyses in cell lines, we checked whether NEST could facilitate the analysis of tumor profiling data. There have been previous studies integrating biological networks with cancer (or disease) biology data to understand the mechanisms of pathogenesis [35, 40–43]. Inspired by these studies, we examined the TCGA tumor profiling data to see whether NEST score computed from gene expression can better predict patient survival than gene expression. For example, over-activation of oncogene *EGFR* is a key feature of Glioblastoma (GBM) [44]. In TCGA GBM profiles [45], while *EGFR* over-expression does not correlate with worse survival (Fig. 4a, Cox-PH *P* value = 0.109), higher *EGFR* NEST score is significantly associated with worse survival (Fig. 4b, Cox-PH *P* value = 0.001).

To systematically evaluate the survival prediction performance, we hypothesized that a good gene-wise survival predictor should show significant higher death risk for oncogenes than for tumor suppressors. We tested this hypothesis on all the annotated oncogenes and tumor suppressors [46] using the TCGA GBM data (Fig. 4c). While gene expression showed no significant difference on survival risk Z-scores, NEST gave significantly higher survival risk for oncogenes than tumor suppressors (Fig. 4c). This observation was corroborated in another independent GBM cohort [47] (Additional file 1: Figure S11), suggesting NEST score to be a much better indicator of GBM survival than gene expression alone. To examine the survival prediction performance of NEST in other cancer types, we used the Wilcoxon rank-sum test to measure the difference of survival risk Z-scores between oncogenes and tumor suppressors. A positive rank-sum Z-score indicates oncogenes with higher survival risk than tumor suppressors, and a negative Z-score indicates the opposite. For low death rate cancers, the Cox-PH survival regression may not get accurate risk estimation for each gene. In contrast, cancer types with high death rate, such as GBM and ovarian cancer (OV), seemed to give positive rank-sum Z-scores that separate oncogenes from tumor suppressors (Fig. 4d). These results suggest that if a gene’s neighbors are over expressed in tumors, the gene itself is more likely to be an oncogene with associated survival risk.

**Fig. 5:**
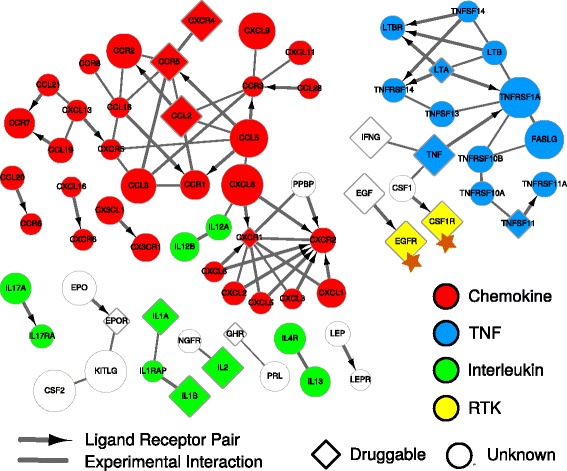
Cytokine receptor interaction network for GBM survival. For all members in KEGG pathway Cytokine Cytokine-Receptor Interaction, we selected the genes indicating death risk with FDR threshold 0.05. The pathway members are colored by their gene family categories, including Chemokine, Tumor Necrosis Factor (TNF), Interleukin, and Receptor Tyrosine Kinase (RTK). A diamond shape indicates the gene to be drug targetable in Drug Bank. The node size is proportional to the NEST score averaged among all GBM patients. Stars are used to label known oncogenes annotated by Vogelstein et al. The directed edge indicates a cytokine-to-receptor relation in KEGG, and undirected edge indicates an experimental protein interaction curated by STRING. The thickness of each edge indicates the interaction confidence

We conducted pathway analysis on all the genes whose NEST scores are associated with GBM survival (FDR < = 0.05), and found ‘cytokine cytokine-receptor interaction’ to be the most enriched KEGG pathway (Additional file 1: Table S2). It was known that cytokines played a pivotal role in the pathogenesis of GBM [48], so we plotted the outcome-associated cytokine genes using CytoScape [49] (Fig. 5). Many of them are known oncogenes in GBM, such as *EGFR* and *CSF1R* [46], and several also have known targeted drugs from Drug Bank [50]. For example, the inhibitors of *EGFR*, *CSF1R*, and *CXCR4* were shown to reduce the invasiveness of Glioma cells or block GBM progression [51–53]. Besides the known druggable genes, many other genes in our prediction could serve as promising targets. For example, NEST predicted *KITLG* as indicator of poor GBM survival which is consistent with the finding that downregulation of *KITLG* inhibits angiogenesis and Glioma growth [54]. Thus, our predictions could sketch a general landscape to investigate therapeutic possibilities for GBM and other cancers.

## Conclusion

To identify distinct features of gene essentiality in CRISPR screens, we developed a network-based method called NEST (Network Essentiality Scoring Tool). We found that essential genes selected in CRISPR screens showed characteristic higher expression level of neighbor genes connected in protein interaction network. Our analysis of Cholera toxin screen in K562 cell also suggests that the quality of CRISPR screen result can be enhanced through the neighbor CRISPR selection score. For a ChIP-seq experiment, NEST can also reliably identify the key TF target genes. Last but not least, NEST score can better predict patient survival than gene expression alone from TCGA tumor profiles. Historically, protein interaction networks were widely used to infer discrete labels such as gene functions, phenotypes [55–57], or gene categories [58]. Our study is different from these previous works in that continuous expression or screen change fold values are integrated with the protein networks. Despite these differences, all of these studies indicate that network information can greatly help biological inference.

NEST significantly outperformed previous methods on gene essentiality prediction and functional screen result enhancement, including all winning methods in the DREAM challenge (Fig. 1f). According to the rule of DREAM challenge, all DREAM methods can gene expression as well as any other features they could utilize. However, NEST outperformed all top DREAM methods. One possible reason is that the gene essentiality gold standard of DREAM is the Achilles shRNA screen data, which is poorly correlated with CRISPR screen (Fig. 1b and Additional file 1: Figure S3A). Because we used CRISPR data as gold standard, those top DREAM methods, optimized to fit Achilles shRNA screen, may not have satisfactory performance.

Several limitations should be noted for our study. NEST computed gene activity is based on network interaction partners, which could have either an activating or a repressive effect. Meanwhile, for compensating interaction such as synthetic lethality, the activation of interaction partners indicates gene loss of function. For example, *PLK1*, an interaction partner of *TP53* in STRING network, was consistently upregulated in cancer cells with inactivated *TP53* compared with those with wild type [59]. We currently summed all neighbor values without distinguishing between activating, repressive, or synthetic lethal relations. Thus, further categorization of network interaction types will be critical for better gene prioritization. Another limitation of our study is that current data on protein interaction network only covered a subset of well-studied genes [60]. Because of the dependence on interaction knowledge, our method may not reliably infer the activity for under-studied genes. As a third limitation, we only tested NEST on gene loss-of-function CRISPR screens. However, for CRISPRa gain of function screen [8, 9], it remains to see whether network-based analysis can bring any predictive power and result enhancement.

In summary, we derived a network-based method, NEST, to interpret and enhance the outcome of genome-wide CRISPR screens, and NEST showed significantly better performance than previous related methods. We recommend researchers using NEST to calculate neighbor CRISPR values from their CRISPR screen result. Moreover, the candidate essential genes in a cell condition might be prioritized before running a large-scale screen to reduce the total number of genes under the screen, which might improve the results and practicality of in vivo CRISPR screens. Besides CRISPR analysis, our method can also identify key targets from ChIP-seq experiments, and find clinical outcome associated genes from tumor profiling data. Thus, we foresee NEST as generally applicable to many applications related with gene essentiality prioritization.

## Materials and methods

### Availability

The web application and source code of NEST are freely available under the GNU Public License v3 at http://nest.dfci.harvard.edu. The source code and testing data of NEST are additionally deposited at https://github.com/foreverdream2/NEST/releases.

### Data collection

For CRISPR screen data, we searched published studies with data publicly available and sgRNA coverage on genome scale for human cell lines until 1 June 2015. There are three studies fulfilling our criterion. In K562 cell, growth phenotype and toxin selection phenotype are screened with CRISPRi technology [8]. In HL60 and A375 cell lines, growth phenotype is screened on genome scale with CRISPR technology [4, 5]. Significant gene hits are called from these datasets by MAGeCK 0.5 with default parameters and FDR threshold 0.05 [29]. For gene essentiality prediction in each cell line, only negatively selected gene hits were considered as gold standard, because most significant gene hits are negatively selected in collected CRISPR experiments (Additional file 1: Figure S1). For gold standard control set, we extracted the same number of genes ranked by MAGeCK on bottom.

For K562, the gene expression profile was downloaded from the Roadmap project [26]. For HL60, the gene expression profile by exon-array was downloaded from the ENCODE project [27] and converted to gene level values by JETTA [61]. For A375, the gene expression profile was downloaded from the CCLE project [28]. For each gene, we normalized the expression value by subtracting the mean across all samples in each cohort. Compared to absolute expression level, the normalized expression value can achieve a better CRISPR prediction performance of NEST (Additional file 1: Figure S12). The TCGA tumor gene expression data was downloaded from TCGA Data Portal on 27 July 2014. Only cohorts that are not embargoed are used. For each cancer cohort, the expression values of all normal control samples were averaged as background, and the difference of gene expression between tumor sample and normal background was analyzed. For NOTCH signaling pathway analysis, the NOTCH off condition is defined as gamma secretase inhibitors (GSI) treatment 3 days, and NOTCH on condition is defined as GSI wash 4 h [62]. The differential expression value between on/off conditions was analyzed [62]. The NOTCH1 ChIP-seq data are generated in our previous work, and the dynamic binding peaks between NOTCH on/off conditions were used [2].

For H3K27ac ChIP-seq profiles, we downloaded data from the Roadmap project [26]. Among all cell lines with CRISPR data collected, K562 is the only one having H3K27ac profile available. Previously, we developed a BETA method to calculate the regulatory potential (RP) on gene promoters from the ChIP-seq profile of a transcription factor or histone modification [36, 37]. We used the implementation in RABIT package with default parameters to calculate the H3K27ac RP scores [39]. For each gene, the RP scores were normalized, by subtracting the mean across all cell lines profiled.

### Network randomization and permutation test

We used stub rewiring method to randomize unweighted STRING network, which preserves gene degree [33]. The edges from each gene are first detached from its partners, and then randomly connected with each other. Since we do not allow self-interaction and duplicated edges, the connection process may fail to finish. In this case, we restart the rewiring process until 98 % edges are reconnected.

Based on random networks, we derived a permutation test to access whether the NEST score of each gene is significantly larger (or smaller) than expected. For each random network, we calculated the NEST values as random NEST. For each gene, we computed the Z-score as (real NEST – average random NEST)/(Stderr of random NEST). If the Z-score is positive, we computed the *P* value as the fraction of random NEST scores that are larger than or equal to the real NEST score. If the Z-score is negative, we computed the *P* values as the fraction of random NEST scores that are smaller than or equal to the real NEST score.

### Survival analysis

We used Cox-PH model to analyze the effect of gene expression or NEST scores on survival. For GBM, there are several factors that affect the survival and we included them as covariates in survival regression, including age, gender, G-CIMP status, and treatment status [45]. So the final survival effect was corrected with the effects of these confounding factors. For TCGA pan-cancer analysis, we only included cancer types with more than 50 patients and 5 % death rate. In the Cox-PH regression, we only included age, gender, and stage (if available) to enable uniform comparison among different cancer types.

## Additional file

Additional file 1:
**Supplementary methods, Supplementary Figures S1 to S12 and Tables S1 and S2.** (PDF 524 kb)
