# Supplementary material for: Network analysis of gene essentiality in functional genomics experiments
Source: Genome Biol. 2015 Oct 30;16:239. doi: 10.1186/s13059-015-0808-9 (PMC4627418; doi:10.1186/s13059-015-0808-9)
Supplement: Additional file 1: — Supplementary methods, Supplementary Figures S1 to S12 and Tables S1 and S2. (PDF 524 kb) [file 13059_2015_808_MOESM1_ESM.pdf]

Supplementary Methods

Method comparison

For gene essentiality prediction, the outcome from CRISPR experiments is used as gold standard. Significant gene hits are called by MAGeCK 0.5 with default parameters and FDR threshold 0.05. Only negatively selected gene hits were considered as gold standard, because most significant gene hits are negatively selected in collected CRISPR experiments (Supplementary Figure S1). For gold standard control set, we extracted the same number of genes ranked on bottom by MAGeCK in negative selection.

For comparison with DREAM, we used the top 3 ranked methods in Sub-challenge 1, where any features can be used for gene essentiality prediction. The best-ranked method used kernel-based representation learning method to reflect dependency among essentiality scores of different genes. This team optimized the kernel and prediction parameter to best reflect the gene-wise similarity in DREAM challenge data. The second ranked method used multiple kernel learning, random forest and kernel ridge representation to boost the ensemble classification accuracy beyond each individual component. This method also incorporated prior knowledge regarding gene interaction networks to improve prediction performance. The third ranked method used Gaussian process regression with carefully tuned parameters to fit the DREAM challenge gene essentiality. For all cell lines included in DREAM challenge, only A375 cell line has CRISPR data collected. So, we only compared the performance on A375 cell.

For comparison with PinnacleZ [1], we only used STRING interactions with confidence score larger than 0.6, because the software crashed after loading the entire STRING network. We took all default parameters in running and grouped all subnetwork markers together as predicted essential genes.

For method of network smoothing, the authors code release (called NBS) depends on an older version of MATLAB 2012 and we cannot run the code with our MATLAB 2015. Thus, we re-implemented the procedure in the same way as described [2] (source code available at: http://nest.dfci.harvard.edu/download/network_smoothing.R).

For comparison with NePhe on CRISPR screen quality enhancement [3], we only used STRING interactions with confidence larger than 0.6, because the software takes more than 60G memory and crash if the whole STRING network is used. Since the input of NePhe is a gene list, we used the negative selection hits from MAGeCK. All default parameters were used in running.

KEGG pathway enrichment

For each gene, we first estimate the study bias of KEGG by counting the number of times that this gene is included in any KEGG pathway annotations. For a give profile of gene scores, we run a logistic regression for each KEGG pathway annotation. The response variable is 1 or 0, indicating whether a gene is annotated in that KEGG pathway. The covariates matrix included the gene scores and gene study bias as background. The Ward test is used to test the significance of given gene scores. The Ward test P-values are converted to FDRs by Benjamini-Hochberg procedure and 0.05 is used as threshold to find significantly enriched KEGG pathways.

Supplementary Figures and Tables

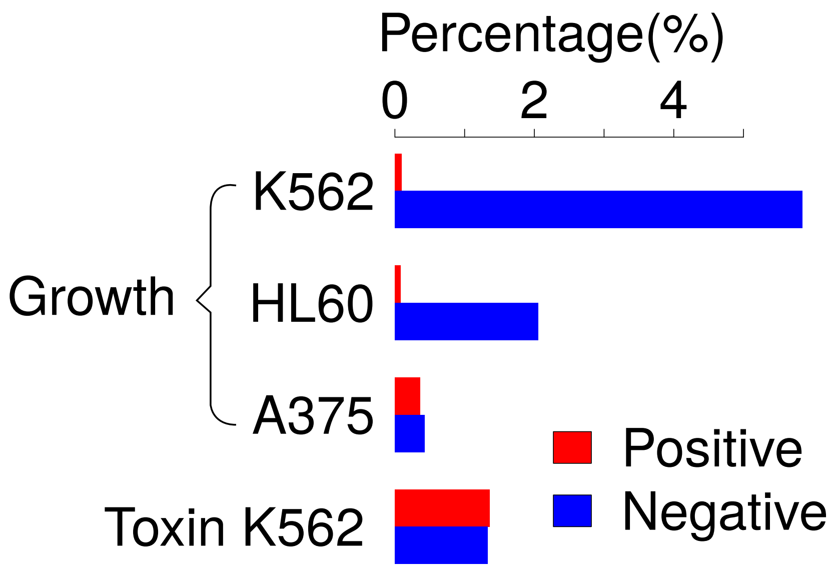

Supplementary Figure S1 - CRISPR screen selection. MAGeCK was used to call significant hits from each CRISPR screen dataset with FDR threshold 0.05. The percentage of significant genes out of all profiled genes is shown for both positive selection and negative selection.

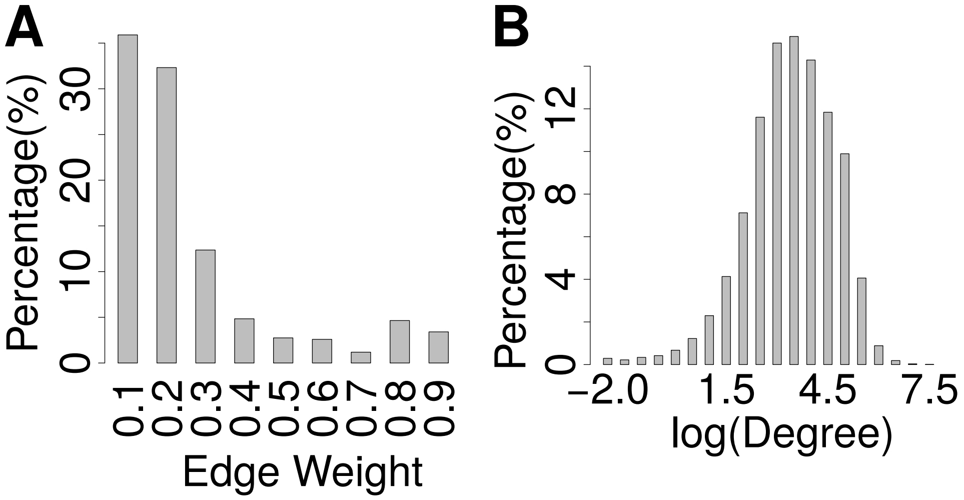

Supplementary Figure S2 - STRING network distribution. (A) The histogram of interaction confidence in STRING network is shown. (B) The histogram of gene degree in STRING network is shown with log scale on x-axis.

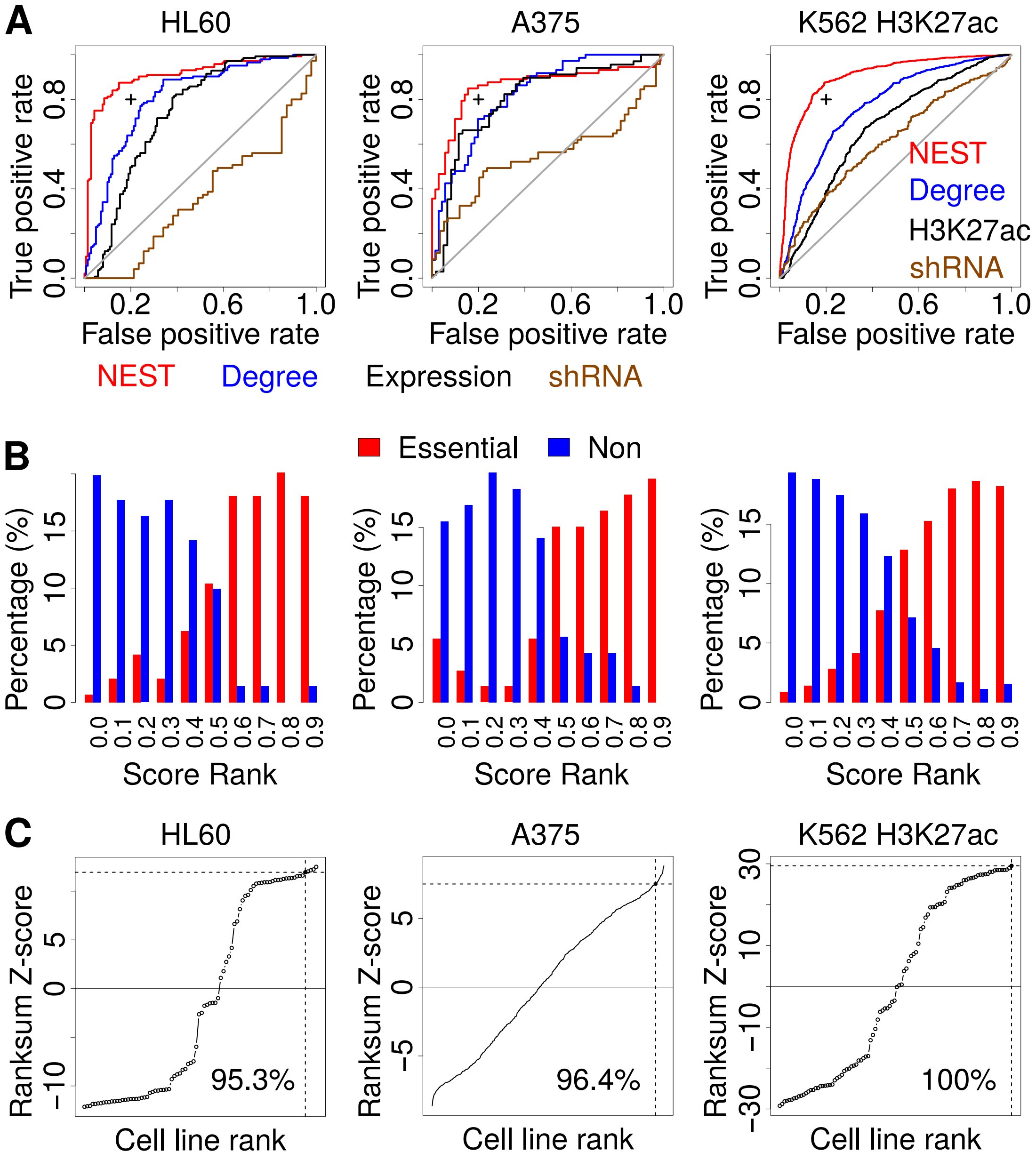

Supplementary Figure S3 - Prediction of CRISPR outcome. (A) ROC curve was used to access the performance of predicting the gene essentiality determined by CRISPR screen in the same way as Figure 1. (B) The NEST scores are converted to relative rank percentiles from 0 to 1. The rank percentile values are shown for essential genes and non-essential genes. (C) The HL60 expression profile was collected from the ENCODE, the A375 expression profile was from the CCLE project and K562 H3K27ac profile was collected from Roadmap project. In each cohort, we calculated the NEST scores for all cell lines profiled and computed their prediction power of gene essentiality in CRISPR screen by Wilcoxon rank-sum test. The rank-sum Z-scores were ranked and the relative rank of each cell line is shown.

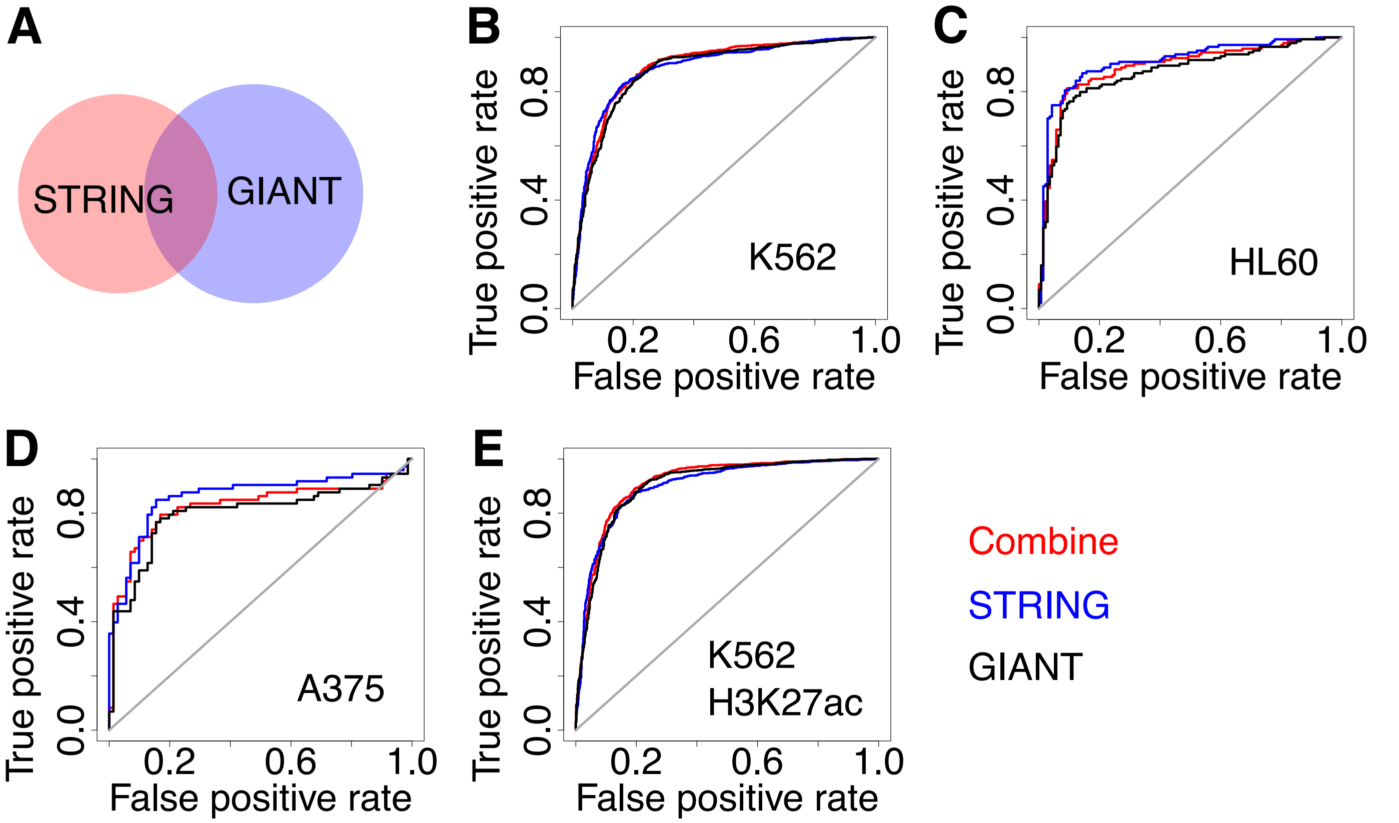

Supplementary Figure S4 - Merge multiple networks. We combined STRING with GIANT network [4]. The all-tissue network of GIANT has about 3.89E7 interactions among 25,689 genes, which is much larger than the STRING network of 2.16E6 interactions among 18,076 genes. (A) Both networks have similar numbers of high confidence interactions with weight $>$ 0.6 (255,130 for STRING, 318,010 for GIANT). Intriguingly for confident interactions, there is very limited overlap between two networks. We combined STRING and GIANT networks by noisy-or on edge weights (weight_combined = 1-(1-weight_STRING)*(weight_GIANT)). If one edge does not exist in either network, we defined its weight as zero. NEST scores are calculated using STRING, GIANT and combined networks. Using CRISPR outcome as gold standard, the prediction performance of NEST score is tested by ROC curves and shown for (B) K562, (C) HL60, (D) A375 and (E) K562 H3K27ac ChIP-seq.

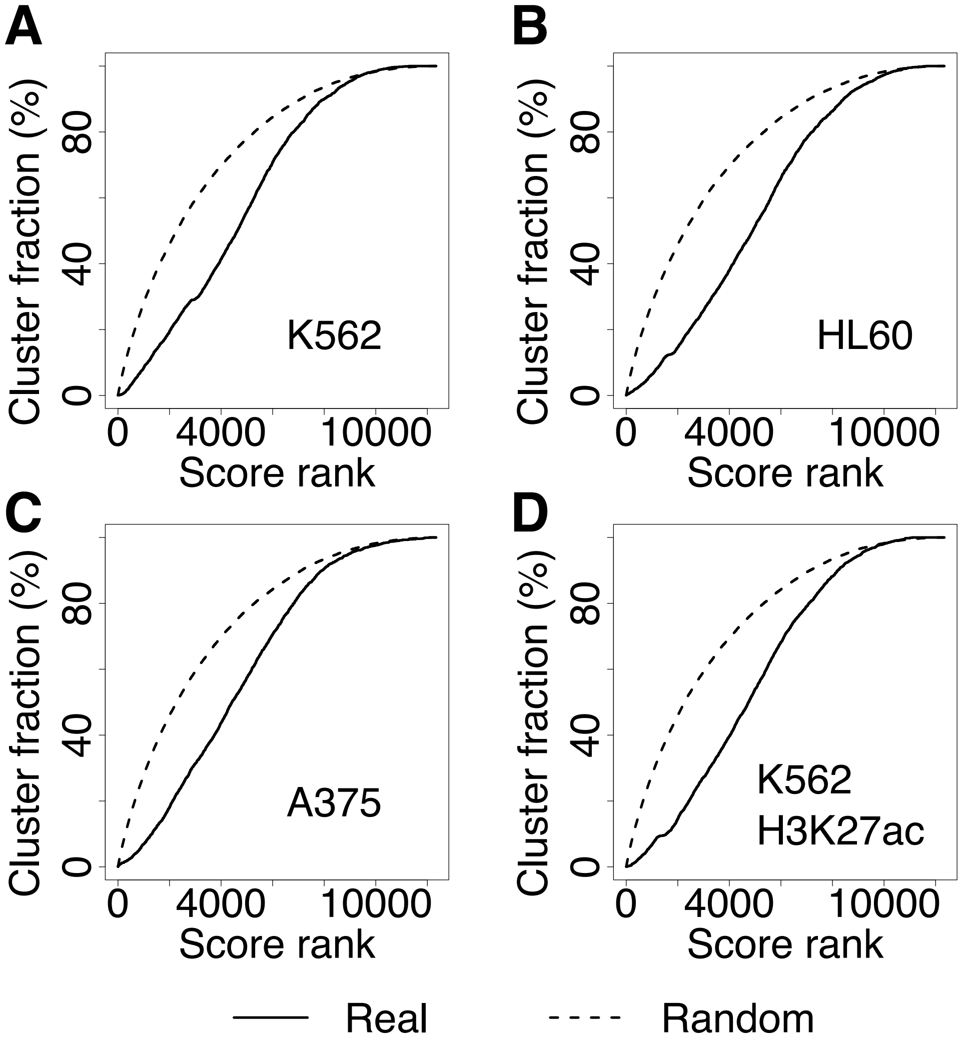

Supplementary Figure S5 - Clustering of NEST scores. The STRING network is clustered by software SPICi to 2271 clusters. In each cohort, all NEST scores were ranked from highest to lowest (X-axis). At each score rank, we counted the fraction of clusters covered by top ranked genes (Y-axis). As random control, we permutated the gene names across all clusters and counted the fraction of clusters in the same way as initial data. Ten randomizations were used to derive the average value curve. The fraction curves are plotted for cohorts (A) K562 expression, (B) HL60 expression, (C) A375 expression and (D) K562 H3K27ac ChIP-seq.

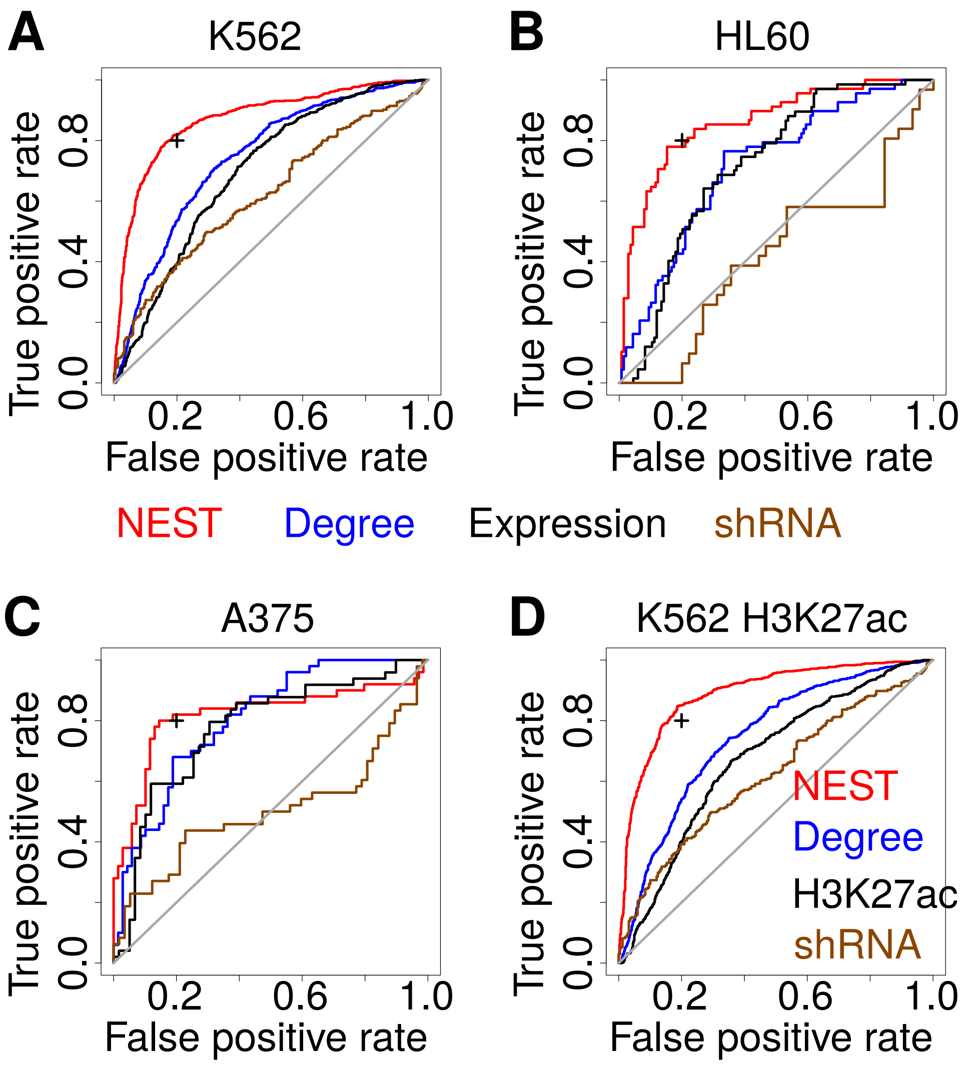

Supplementary Figure S6 - Performance after housekeeping gene removal. The annotation of human house keeping genes is collected from Eisenberg et al [5], plus all genes included in KEGG pathway Ribosome, Proteasome, Spliceosome, DNA replication and RNA polymerase. We removed housekeeping genes from our analysis and tested the prediction performance of NEST scores on CRISPR screen data. The ROC curves are shown for (A) K562, (B) HL60, (C) A375 and (D) K562 H3K27ac.

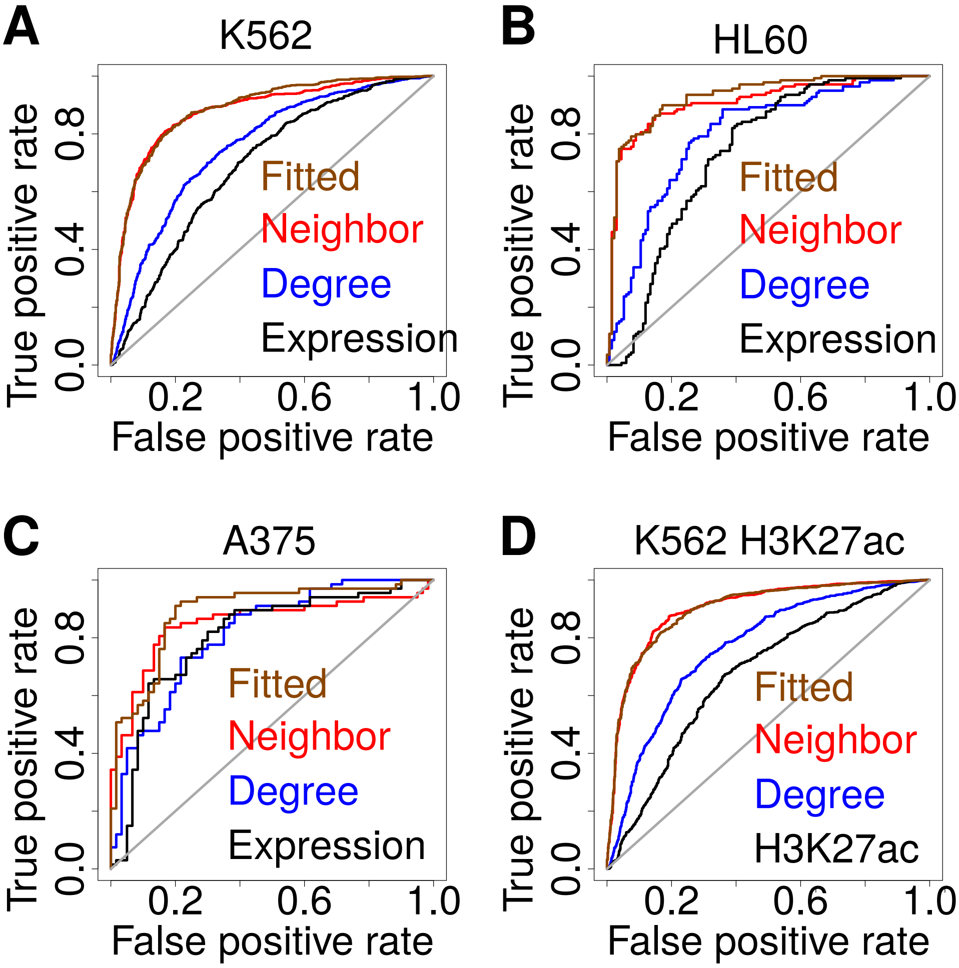

Supplementary Figure S7 - Performance of fitted gene essentiality value. For each CRISPR screen, we applied logistic regression with response variable as CRISPR outcome and covariates as gene expression, neighbor gene expression (NEST score), and network degree (Table 1). Based on the above logistic regression, the essentiality probability is predicted for each gene as fitted value, which combined effects from all covariates. Using CRISPR outcome as gold standard, the prediction performance is tested by ROC curves and shown for (A) K562, (B) HL60, (C) A375 and (D) K562 H3K27ac.

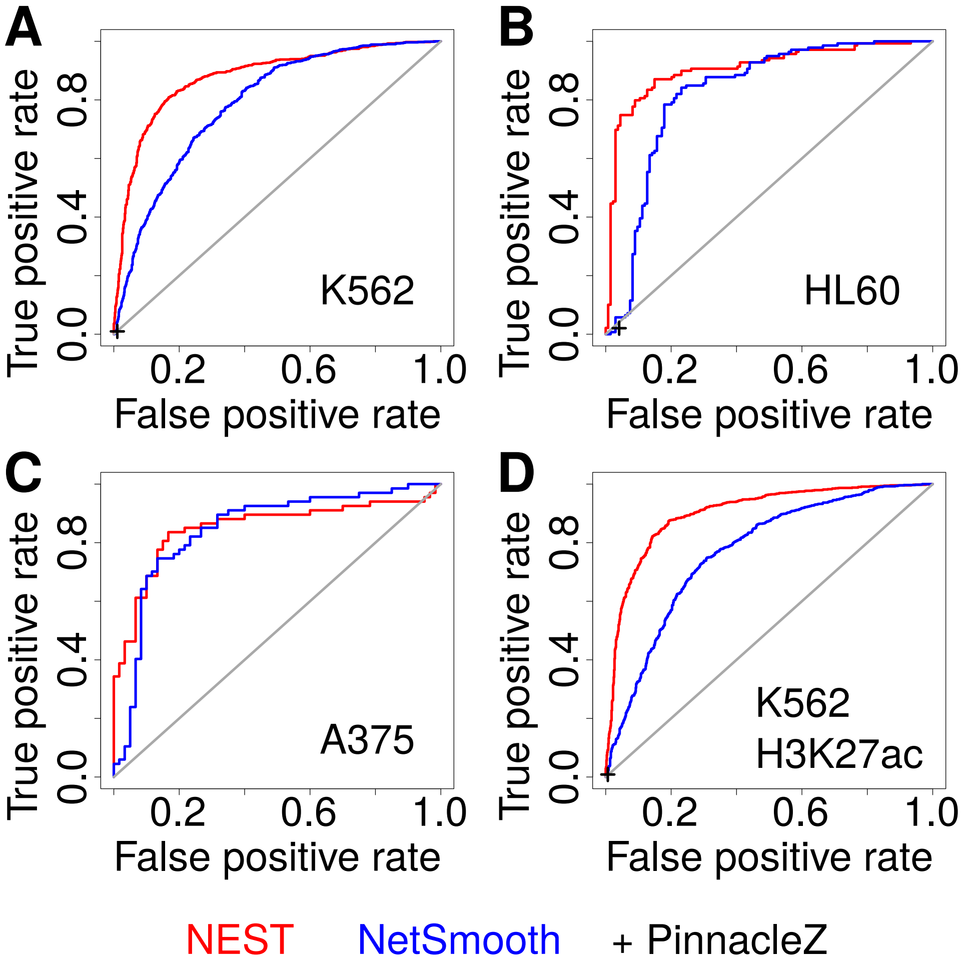

Supplementary Figure S8 - Method Comparison on CRISPR prediction. Using essential genes selected in CRISPR screen as gold standard, the prediction performance is compared among NEST, Network Smoothing [2] and PinnacleZ [1]. Because the outcome of PinnacleZ is a set of genes (subnetwork markers), only one point (black cross dot) is shown in ROC curve. For A375 cell with gene expression measured by CCLE project, the PinnacleZ software failed to produce any result. Thus, no performance point was included in the A375 ROC curve. The ROC curves are shown for (A) K562, (B) HL60, (C) A375 and (D) K562 H3K27ac.

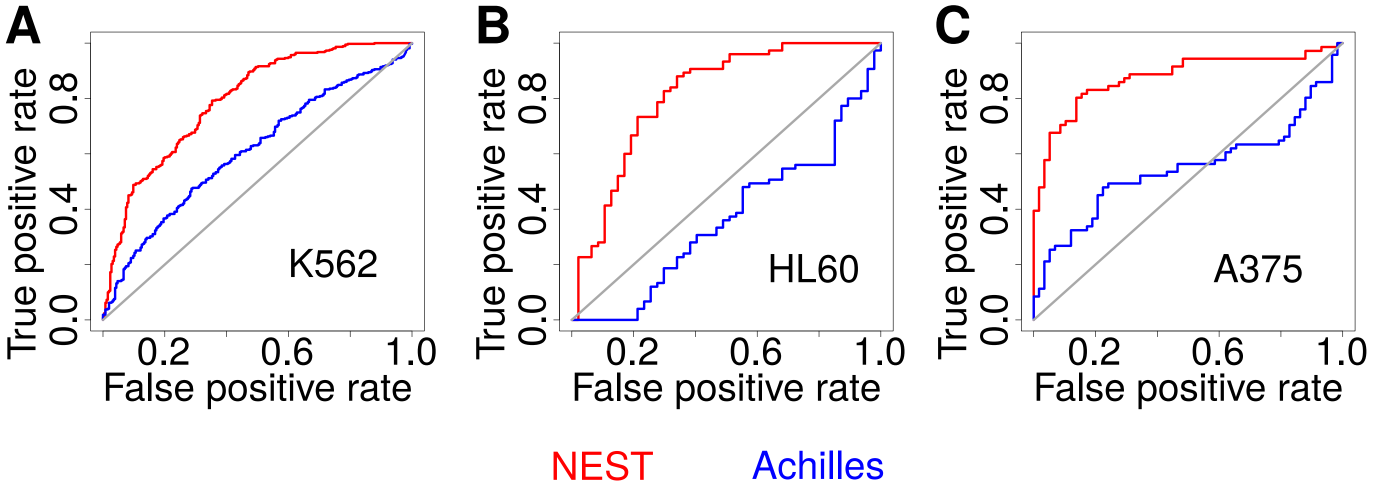

Supplementary Figure S9 - Enhancement of shRNA screen result. For gene, neighbor shRNA screen score was computed with NEST in different cell lines. Each gene may have several fold change values from different shRNAs in screen design, and the median value was used. The prediction performance on CRISPR outcome is compared between NEST scores and initial screen scores from the Achilles project for (A) K562, (B) HL60 and (C) A375 cells.

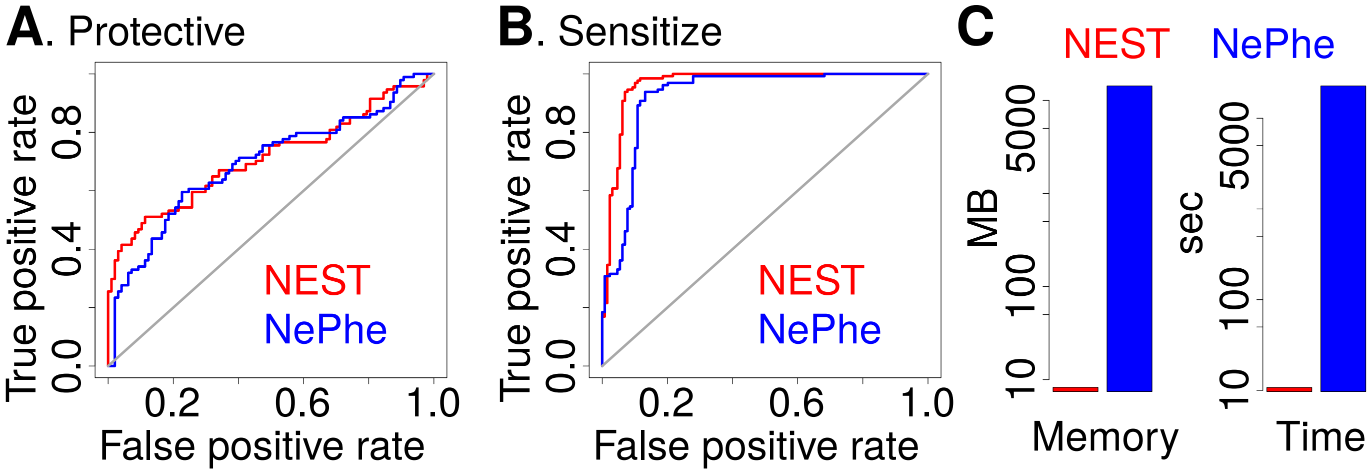

Supplementary Figure S10 - Method comparison on CRISPR enhancement. For K562 Cholera toxin CRISPRi screen data, we applied both NEST and NePhe for quality enhancement and compared their prediction performance in the same way as Figure 2AB. ROC curves are shown for gold standard set of (A) protective genes and (B) sensitizing genes. (C) The time and memory usage is compared.

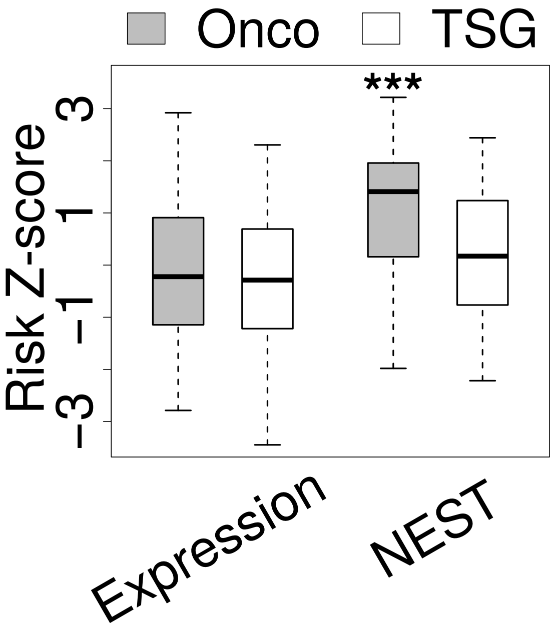

Supplementary Figure S11 - Prediction of GBM patient survival. The analysis was done in the same way as Figure 4C in main text. The GBM patient expression and clinical data are from Gavendeel et al [6]. For each gene, we calculated a death risk Z-score by Cox-PH model from either gene expression or NEST scores. We compared the distribution of Z-scores for oncogenes (Onco) and tumor suppressor genes (TSG) based on the annotation from Vogelstein et al [7]. The bottom and top of the boxes are the 25th and 75th percentiles (interquartile range). Whiskers on the top and bottom represent the maximum and minimum data points within the range represented by 1.5 times the inter-quartile range. The distribution of Z-scores is compared between Oncogene and TSG category by Wilcoxon rank-sum test and three stars represents P-value $<$ 0.001.

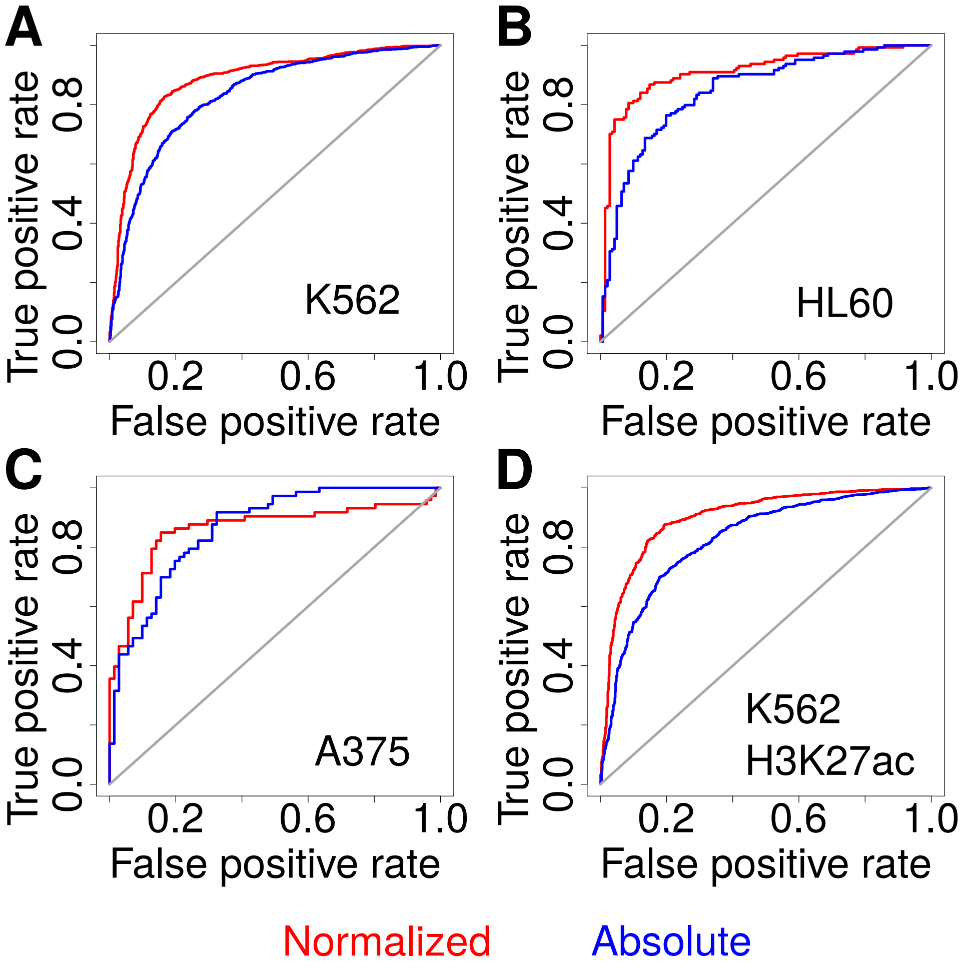

Supplementary Figure S12 - gene expression normalization. CRISPR prediction performance of NEST scores is compared by ROC curves between results using normalized expression and absolute expression. The ROC curves are shown for (A) K562, (B) HL60, (C) A375 and (D) K562 H3K27ac ChIP-seq.

	K562	HL60	A375
K562		0.075	0.032
HL60	0.075		0.048
A375	0.032	0.048	

Supplementary Table S1 - Overlap of negative selection. The overlap between negatively selected gene hits are shown by Jaccard coefficients between all pairs of cell lines. Jaccard coefficient between set A and B is defined as $|A \cap B|/(|A| + |B| - |A \cap B|)$. So, value 0 means no overlap and value 1 means perfect overlap.

KEGG Pathway	Percentage(%)	Z	Pubmed ID
CYTOKINE CYTOKINE RECEPTOR INTERACTION	68.84	13.68	22075379
HEMATOPOIETIC CELL LINEAGE	91.12	8.73	
NATURAL KILLER CELL MEDIATED CYTOTOXICITY	60.36	8.28	19265131
JAK STAT SIGNALING PATHWAY	77.51	8.07	22879070
TOLL LIKE RECEPTOR SIGNALING PATHWAY	88.76	7.83	25411122

Supplementary Table S2 - KEGG pathway enrichment for GBM death risk genes. We did two types of KEGG pathway enrichment analysis for TCGA GBM. In type one analysis, we analyzed the enriched KEGG pathway by the NEST scores for each GBM tumor sample by logistic regression. The significantly enriched pathways were selected with FDR threshold 0.05, and only pathways enriched in more than 60% tumors were reported. In type two analysis, the survival risk Z-scores are analyzed for enriched of KEGG pathways by logistic regression and enriched pathways are selected with FDR threshold 0.05. The overlap between two types of analysis is considered, and we only show the top five enriched KEGG pathways ranked by survival risk Logit Z-score. Pubmed IDs are attached for any literature supports of enriched KEGG pathways.

References

[1] Chuang HY, Lee E, Liu YT, Lee D, Ideker T: **Network-based classification of breast cancer metastasis**. *Mol Syst Biol* 2007, **3**:140.

[2] Hofree M, Shen JP, Carter H, Gross A, Ideker T: **Network-based stratification of tumor mutations**. *Nat Methods* 2013, **10**(11):1108–15.

[3] Wang L, Tu Z, Sun F: **A network-based integrative approach to prioritize reliable hits from multiple genome-wide RNAi screens in Drosophila**. *BMC Genomics* 2009, **10**:220.

[4] Greene CS, Krishnan A, Wong AK, Ricciotti E, et al.: **Understanding multicellular function and disease with human tissue-specific networks**. *Nat Genet* 2015, **47**(6):569–76.

[5] Eisenberg E, Levanon EY: **Human housekeeping genes are compact**. *Trends Genet* 2003, **19**(7):362–5.

[6] Gravendeel LA, Kouwenhoven MC, Gevaert O, French PJ, et al.: **Intrinsic gene expression profiles of gliomas are a better predictor of survival than histology**. *Cancer Res* 2009, **69**(23):9065–72.

[7] Vogelstein B, Papadopoulos N, Velculescu VE, Zhou S, Diaz LAJ, Kinzler KW: **Cancer genome landscapes**. *Science* 2013, **339**(6127):1546–58.
